# Identifying vulnerable populations in the electronic Framingham Heart Study to improve digital device adherence

**DOI:** 10.1038/s41746-023-00789-9

**Published:** 2023-03-13

**Authors:** Mirja Mittermaier, Kaushik P. Venkatesh, Joseph C. Kvedar

**Affiliations:** 1 Charité—Universitätsmedizin Berlin, Corporate Member of Freie Universität Berlin and Humboldt-Universität zu Berlin, Department of Infectious Diseases, Respiratory Medicine and Critical Care, Berlin, Germany; 2 Berlin Institute of Health at Charité—Universitätsmedizin Berlin, Charitéplatz 1, 10117 Berlin, Germany; 3 Harvard Medical School, Boston, MA USA

**Keywords:** Diagnosis, Developing world

## Abstract

The usage of digital devices in clinical and research settings has rapidly increased. Despite their promise, optimal use of these devices is often hampered by low adherence. The relevant factors predictive of long-term adherence have yet to be fully explored. A recent study investigated device usage over 12 months in a cohort of the electronic Framingham Heart Study. It identified sociodemographic and health-related factors associated with the long-term use of three digital health components: a smartphone app, a digital blood pressure cuff, and a smartwatch. The authors found that depressive symptoms and lower self-rated health were associated with lower smartwatch usage. Female sex and higher education levels were associated with higher app-based survey completion. Here, we discuss factors predictive for adherence and personalized strategies to promote adherence to digital tools.

## Digital device adherence

Digital devices like smartwatches, fitness trackers, and digital medical devices such as digital blood pressure (BP) cuffs and spirometers have become increasingly common. These devices offer new ways to track patient health in real-time and facilitate self-management and prevention of disease. Such digital health innovations could also transform clinical trials, including prospective cohort studies or randomized controlled trials (RCTs), through new dimensions of data collected longitudinally outside a clinical setting.

For most applications, daily and long-term use of digital devices is required to collect meaningful data for clinical and research contexts. In clinical practice, long-term adherence is essential to the quality of care provided. For example, endocrinologists and primary care physicians receive real-time glucose data for insulin management. Some tests (such as those for hypoglycemia) require continuous monitoring during a specific time window. While clinical trials have found that adherence to digital devices is often low^1,2^, most studies evaluating such adherence behaviors have captured data for only a short period. Further work is needed to explore the factors associated with long-term usage.

Pathiravasan et al. aimed to identify factors associated with using digital devices in a nested e-cohort (eFHS) embedded within the well-known Framingham Heart Study^3^. Usage of digital components, i.e., smartphone app, digital BP cuff, and smartwatch, was observed over 12 months. The authors identified several sociodemographic and health-related factors associated with long-term usage by applying multivariate analysis.

Depressive symptoms and lower than excellent self-rated health were associated with lower use of the smartwatch^3^. This corresponds with other studies showing digital devices are least used by high-risk patients such as elderly persons and patients with chronic diseases^4^. Further, previous studies have shown that chronic diseases, lower self-reported health, and depression were associated with lower usage of wearing activity trackers^5,6^. This data helps to identify patient subgroups who might benefit from additional measures to support the use of digital devices to promote health. Identifying each patient’s unique needs is crucial in encouraging adherence to technology. For example, patients unable to track medication intake using an app may benefit from a pill that monitors ingestions^7^ or an electronic medication bottle cap with audio-visual reminder^8^.

On the other hand, certain health and socioeconomic factors determine good adherence to digital medical devices. For example, Pathiravasan et al. found that higher levels of education and female sex were associated with higher completion of the app-based surveys. They also found that iPhone users were likelier to submit app-based surveys than Android users. Android users were less likely to be women and had lower educational and health levels. A previous study^9^ showed that iPhone users are more tech-enabled and interested in retail-mobile apps. This suggests that the type of smartphone itself can be a proxy for socioeconomic and educational factors associated with digital device usage^3^. It also suggests that developers and health promotion leaders should be keen to avoid entrenching disparities by targeting multiple device platforms.

Digital health devices can empower older patients to maintain functional independence by self-managing their health conditions using symptom trackers and regular monitoring. Elderly patients face significant barriers (e.g., digital literacy) that affect their ability to interact with digital devices, a phenomenon known as the digital divide^10^. However, Pathiravasan et al^3^. found that older adults, once enrolled, were more likely to engage with digital devices than younger participants, consistent with previous findings^11^. These findings support investment into digital literacy for elderly patients, which can be delivered by individual physicians, provider groups, community organizations, etc. It also encourages further action to include elderly populations in digital health initiatives rather than exclude them due to starting hurdles.

While digital devices offer immense potential for personalized medicine, they also provide new ways to address population-based impact. Daily habits can be challenging to change, and digital devices are more likely to be used if they are easily integrated into a patient’s lifestyle. A person who has never worn a watch might find it harder to wear a smartwatch daily. A diabetes patient habituating to measuring blood sugar several times per day might easily switch to a device that automatically saves and transfers data. In these ways, digital health tools should be offered and integrated into patients’ lives based on their unique behavioral, environmental, health, and social contexts.

## Conclusion

Altogether, digital devices have an immense potential to transform clinical delivery and clinical research. To fully exploit the potential of digital devices across all populations, it is pivotal to identify the complex factors contributing to and preventing adherence. Developing personalized supporting strategies for identified patient subgroups is essential to increase digital device adherence.
